# Parental Education and Youth Inhibitory Control in the Adolescent Brain Cognitive Development (ABCD) Study: Blacks’ Diminished Returns

**DOI:** 10.3390/brainsci10050312

**Published:** 2020-05-21

**Authors:** Shervin Assari

**Affiliations:** Department of Family Medicine, Charles R Drew University of Medicine and Science, Los Angeles, CA 90059, USA; assari@umich.edu; Tel.: +734-232-0445; Fax: +734-615-8739

**Keywords:** race/ethnicity, ethnicity, socioeconomic status, youth, cognition, brain, inhibitory control

## Abstract

Background: Non-Hispanic Black (NHB) youth are at a higher risk of high-risk behaviors compared to non-Hispanic White (NHW) youth. Some of this racial gap is shown to be due to weaker effects of parental educational attainment on reducing the prevalence of behavioral risk factors such as impulsivity, substance use, aggression, obesity, and poor school performance for NHBs, a pattern called Minorities’ Diminished Returns. These diminishing returns may be due to lower than expected effects of parental education on inhibitory control. Aim: We compared NHW and NHB youth for the effect of parental educational attainment on youth inhibitory control, a psychological and cognitive construct that closely predicts high-risk behaviors such as the use of drugs, alcohol, and tobacco. Methods: This was a cross-sectional analysis that included 4188 youth from the Adolescent Brain Cognitive Development (ABCD) study. The independent variable was parental educational attainment. The main outcome was youth inhibitory control measured by the stop-signal task (SST), which was validated by parent reports on the Child Behavior Checklist (CBCL). Results: In race/ethnicity-stratified models, high parental educational attainment was associated with a higher level of inhibitory control for NHB than NHW youth. In the pooled sample, race/ethnicity showed a statistically significant interaction with parental educational attainment on youth inhibitory control suggesting that high parental educational attainment has a smaller boosting effect on inhibitory control for NHB than NHW youth. Conclusion: Parental educational attainment boosts inhibitory control for NHW but not NHB youth. To minimize the racial gap in youth brain development, we need to address societal barriers that diminish the returns of family economic and human resources, particularly parental educational attainment, for racial and ethnic minority youth. Social and public policies should address structural and societal barriers such as social stratification, segregation, racism, and discrimination that hinder NHB parents’ abilities to effectively mobilize their human resources and secure tangible outcomes for their developing youth.

## 1. Introduction

Inhibitory control (IC), also known as response inhibition, is a cognitive process/executive function—that can be defined as an ability to inhibit impulses in order to increase the chance of appropriate behaviors that are consistent with completing long-term goals [1,2]. An extreme example of a disorder with impaired IC is attention deficit hyperactivity disorder (ADHD) [3]. Low IC is associated with a wide range of outcomes such as obesity, food choices, eating disorders, school performance, peer preferences, externalizing behaviors, aggression, prosocial behaviors, sexual debut, and use of drugs, alcohol, and tobacco [4,5,6,7,8,9,10,11,12]. Low IC may be one of the reasons why youth from low socioeconomic status (SES) and racial and ethnic minority groups engage in more risky behaviors, compared to high SES and majority youth [1,13,14,15,16]. Thus, studies are needed that investigate the additive effects of race/ethnicity and SES on youth IC as a mechanism of disparities and inequalities in high-risk behaviors [1,10,17,18,19]. 

Family SES and parental behaviors are shown to be predictors of IC in youth [20,21,22,23,24]. In a study on 147 7–10-year-old children, Cabello et al. investigated the relationship between parental educational attainment and youth IC as well as aggressive behavior. Teachers rated the aggressive behavior of the children using the Teacher Rating Scale (TRS) of the Behavior Assessment System for Children 2 (BASC-2). The youth themselves completed a go/no-go task that assessed their IC. Parents reported their educational attainment. Analysis of the data showed that both lower parental educational attainment and lower IC were predictive of higher aggressive behavior in the youth. Interestingly, authors found evidence suggesting that IC partially mediates the effects of parental educational attainment on aggressive behavior. However, sex moderated this mediation, as IC explained this effect for boys, not girls. Their study suggests the parental educational attainment impacts IC and IC may be why parental educational attainment is associated with youth aggressive behavior, particularly in boys [6].

Compared to non-Hispanic White (NHW) youth, non-Hispanic Black (NHB) youth are at an increased risk of high-risk behaviors that are largely associated with poor IC. For example, NHB youth are more likely than NHW youth to be at risk of engaging in aggressive behavior [25], early sexual debut [26], and becoming school dropouts [27]. As many of these early undesired outcomes are gateways to future economic and health problems later in life [28,29,30,31], it is important to study why and how IC is different in NHB and NHW youth. Such knowledge may be helpful in closing, or at least reducing, the existing racial and ethnic economic and health inequalities experienced later in life [28,29,30,31].

Given the close overlap between race/ethnicity and SES in the US, there has been an interest in trying to decompose the effects of race/ethnicity and SES on health and behavioral inequalities [32,33,34]. In theory, both low SES and racial/ethnic minority status generate marginalization and increase exposure to food and housing insecurity, economic adversities, stress, and financial difficulties [35,36,37,38]. Thus, one remaining question is whether race/ethnicity and SES have separate, additive, or multiplicative effects on health inequalities [39,40,41,42]. While SES provides access to buffers and protections, the SES effects on health and behaviors and their precursors [39,40,41,42] may depend on race/ethnicity. In addition, SES may carry the effect of race/ethnicity on health and behavioral outcomes [43]. 

There are, however, two complementary hypotheses that explain how race/ethnicity and family SES may jointly impact youth health and behavioral outcomes. The first hypothesis, a more traditional one, attributes the racial and ethnic gap in youth outcomes to racial and ethnic differences in family SES (e.g., lower SES in NHBs than NHWs) [43,44,45,46]. In this view, family SES is believed to mediate the effects of racial and ethnic minority status on youth outcomes [47,48,49]. As such, the belief is that enhancing racial and ethnic minorities’ family SES and closing the racial and ethnic differences in SES would be the primary strategy for closing racial and ethnic youth inequalities [50,51]. Some policy solutions based on this view is income redistribution, income tax credit, and empowering racial and ethnic minorities to attain and accumulate education, income, and wealth.

The alternative explanation is Minorities’ Diminished Returns (MDRs) [52,53], which explains that some of the racial and ethnic inequalities are due to the weaker effects of family SES indicators for NHBs than NHWs. This view is supported by extensive empirical evidence showing that parental education [54], family income [55,56], and marital status [57] generate more outcomes for NHW than NHB youth. However, this literature is mainly focused on self-reported outcomes [54,55,56,58,59]. For example, high family SES showed smaller preventive effects on impulsivity [55], ADHD [60], depression [58], anxiety [61], aggression [54], poor GPA [54,62,63], and substance use [54] for NHB than NHW youth. That means, while high SES NHW youth show the lowest levels of risk across domains, high SES NHB youth remain at high levels of risk across multiple domains and outcomes. 

As shown by the MDRs literature, parental educational attainment [64,65,66] generates unequal outcomes for diverse racial and ethnic groups. This might be because society may differently allow NHB and NHW parents to mobilize their education and secure high paying jobs, particularly at high levels of education [53,55,61,65,67,68]. As a result of diminishing returns of parental education, compared to their non-HW counterparts, NHB youth with highly educated parents may show worse than expected outcomes that are disproportionate to their family SES [52,53,55,56,59]. 

## 2. Aims

To extend the existing knowledge on the role of IC as a mechanism for explaining MDRs for other behavioral outcomes, we studied the interactive and combined effects of race/ethnicity and parental educational attainment on youth outcomes. This study compared NHB and NHW youth for the effects of parental educational attainment, as one of the strongest family SES indicators, on youth IC. We expected weaker effects of parental educational attainment on youth IC for NHB than NHW youth. 

## 3. Methods

### 3.1. Design and Settings

We performed a secondary analysis of data from the Adolescent Brain Cognitive Development (ABCD) study [69,70,71,72,73], a landmark youth brain development study in the United States. Detailed information on the details of the ABCD study is available elsewhere [69,74].

### 3.2. Participants and Sampling

Participants of the ABCD study were youth age 9–10 years. Youth in the ABCD study were recruited from multiple cities across states. Overall, there were 21 sites that recruited youth to the ABCD study. The recruitment of the ABCD sample was mainly done through school systems. A detailed description of the ABCD sampling is available here [75]. Four thousand one hundred eighty-eight participants entered our analysis. Eligibility for our analysis had valid data on race/ethnicity, CBCL, task-based IC, parental education, marital status, and being NHB or NHW. 

### 3.3. Study Variables 

The study variables included race/ethnicity, demographic factors, parental educational attainment, parental marital status, and task-based IC (validated by the Child Behavior Checklist (CBCL).

#### 3.3.1. Outcome

The study also used the stop-signal task (SST) to measure IC. The SST applied two runs of 180 trials showing images of a black arrow pointing either right or left are displayed on the screen participants’ view while in the scanner. They were instructed to click the appropriate button corresponding to the arrow direction as quickly as they can after seeing the image using their dominant hand. Thirty of the 180 trials display neither option, signaling the participant to inhibit answering with either option being randomly dispersed throughout the trials. Successful inhibition of motor response represents a successful trial, while impulsively answering with either wrong answer is considered unsuccessful inhibition. IC was measured as the rate of correct "Stop" trials in the run. This variable was treated as a continuous measure with a higher score indicating a higher level of IC [76,77,78,79]. 

#### 3.3.2. Validation of the IC Using CBCL Domains

We used the Child Behavior Checklist (CBCL) to validate lab(task) based measures of IC. The CBCL, also known as the Achenbach System of Empirically Based Assessment, the study had the following eight outcomes: (1) Anxious and depressed mood, (2) withdrawn and depressed affect, (3) social and interpersonal problems, (4) somatic complaints, (5) thought problems, (6) attention problems, (7) violent and aggressive behaviors, and (8) rule-breaking behaviors [80]. These CBCL sub-scores closely correlate with the Diagnostic and Statistical Manual of Mental Disorders (DSM-IV-TR) based diagnoses [81]. The CBCL instrument uses parental reports to screen for social, behavioral, and emotional problems. The CBCL is commonly used across settings including but not limited to schools, medical settings, mental health facilities, child and family services, Health Management Organizations, and public health agencies [82]. CBCL has been used by thousands of published scholarly articles [82]. As expected, in our study, IC was correlated with behavioral rather than emotional domains of the CBCL, which was an indicator of its validity. 

#### 3.3.3. Moderator

*Race/ethnicity*. Race/ethnicity was self-identified. Race/ethnicity was a categorical variable and coded 1 for NHBs and 0 for NHWs (reference category). 

#### 3.3.4. Independent Variable

*Parental Educational Attainment.* Participants were asked, “What is the highest grade or level of school you have completed or the highest degree you have received?” Responses were 0 = Never attended/Kindergarten only; 1 = 1st grade; 2 = 2nd grade; 3 = 3rd grade; 4 = 4th grade 4; 5 = 5th grade; 6 = 6th grade 6; 7 = 7th grade 7; 8 = 8th grade; 9 = 9th grade; 10 = 10th grade 10; 11 = 11th grade; 12 = 12th grade; 13 = High school graduate; 14 = GED or equivalent Diploma; 15 = Some college; 16 = Associate degree: Occupational; 17 = Associate degree: Academic Program; 18 = Bachelor’s degree (ex. BA; 19 = Master’s degree (ex. MA; 20 = Professional School degree (ex. MD; 21 = Doctoral degree. This variable was an interval measure with a range between 1 and 21, with a higher score indicating higher educational attainment.

#### 3.3.5. Confounders

Age, sex, and parental marital status were the confounders. Parents reported the age of their youth. Age (years) was calculated as the difference between the date of birth to the date of enrollment to the study. Sex was a dichotomous variable: males = 1, females = 0. Parental marital status was a dichotomous variable. This variable was self-reported by the parent who was interviewed. This variable was coded as married = 1 vs. other = 0.

### 3.4. Data Analysis 

We used the statistical package SPSS to perform our data analysis. Mean (standard deviation (SD)) and frequency (%) were described depending on the variable type. We also performed a Pearson bivariate test to test bivariate associations between study variables. For our multivariable modeling, we performed four linear regression models. Our first two models were performed specifically to race/ethnicity (race-stratified models). *Model 1* was tested in NHW, and *Model 2* was tested in NHB youth. The next two models were performed in the overall sample. *Model 3* was performed without the interaction terms. *Model 4* added an interaction term between race/ethnicity and parental education attainment. Our models used age, sex, and marital status were the covariates. Unstandardized regression coefficient (b), standardized regression coefficient, SE, 95% CI, and *p*-value were reported for each model. *p* values equal to or less than 0.05 were statistically significant.

### 3.5. Ethical Aspect

The ABCD study received an Institutional Review Board (IRB) approval from the University of California, San Diego (UCSD). Each youth participant provided an assent. Each parent signed an informed consent [74]. As this analysis was performed on fully de-identified data, the study was found to be non-human subject research. Thus, our analysis was exempt from a full IRB review.

## 4. Results

### 4.1. Descriptives

As shown in Table 1, 4188, 8–11 years old youth entered to this analysis. From this number, most were NHWs (*n* = 2985; 71.3%) and the rest were NHBs (*n* = 1203; 28.7%). Table 1 presents a summary of the descriptive statistics for the pooled sample. 

Table 2 shows a correlation matrix of all study variables, including the CBCL reports in the pooled sample and by race/ethnicity. In the pooled sample, NHB status was closely associated with lower parental education and marital status but was not associated with IC. IC was associated with behavioral rather than emotional domains of CBCL measure, which indicated the validity of our IC measure. In the pooled sample, parental educational attainment was not correlated with IC. In NHWs (r = 0.06, *p* < 0.05), but not in NHBs (r = −0.04, *p* > 0.05), parental educational attainment was correlated with IC.

### 4.2. Multivariate Analysis (Race-Stratified models)

Table 3 shows the results of two linear regression models in racial/ethnic groups. *Model 3* (NHW) showed protective effects of high parental educational attainment on IC of NHW youth. *Model 4* (NHB) did not have a protective effect of high parental educational attainment on IC for NHB youth.

### 4.3. Multivariate Analysis (Pooled Sample)

Table 4 shows the results of two linear regression models in the overall (pooled) sample. *Model 1* (Main Effect Model) showed an only marginally significant effect of high parental educational attainment on IC. *Model 2* (Interaction Model) showed a statistically significant interaction term between race/ethnicity and parental educational attainment on IC, suggesting that the boosting effect of high parental educational attainment on IC is significantly weaker for NHB youth relative to NHW youth.

## 5. Discussion

In our race-specific models, high parental educational attainment was associated with higher IC for NHW but not NHB youth. This finding was also supported by a statistical interaction between race/ethnicity and parental educational attainment on youth IC in the pooled sample. Thus, high parental educational attainment has smaller boosting effects on IC for NHB than NHW youth. 

Here first, we discuss the observed MDRs. That is why NHB and NHW youth show a different gain in terms of increased IC due to an increase in parental educational attainment. In this section, we report similar literature and also some potential societal mechanisms that may help us understand these MDRs. Then we discuss the results specific to the IC. That is, SES (parental education) boosts IC and, at the same time, race (NHB) may reduce IC. However, the association between race and IC could only be observed for parental reports rather than the lab-based measure of IC. In the end, we list the limitations and strengths of our study. Among the strengths, we discuss why there were different methods of measuring IC (based on the IC neurocognitive task and parent’s behavioral ratings). 

The observed diminished returns of parental education on IC is in line with the MDRs reported on impulsivity [55], ADHD [60], depression [58], anxiety [61], aggression [54], GPA [54,62,63], and substance use [54]. A large body empirical evidence supports diminishing returns of parental and own educational attainment for NHBs than NHWs [67,83,84,85]. MDRs are already documented both within individuals and families, and hold across SES resources, age groups, outcomes, and marginalizing identities [52,53]. MDRs are shown for income [55], education [84], employment [86], and marital status [61]. Parental educational attainment results in more gain for NHW than NHB youth [55,56,59], adults [67], and older adults [87]. Also, MDRs not only apply to NHBs [56], or HWs [84,88,89,90] but also for Asian Americans [91], Native Americans [92], and LGBTQ persons [83].

Various mechanisms may be involved in explaining the MDRs of parental educational attainment or NHB families. NHB families face disproportionately higher levels of financial, environmental, and race-related stress in their daily lives, at all SES levels. According to the social reproduction theory, intergenerational educational outcomes may vary across groups [93]. Chetty showed that the intersection of race/ethnicity and sex alter the likelihood of upward social mobility in the US [94]. Increased exposure to stress is believed to reduce youths’ ability to gain from their available SES resources such as education. It is shown that for NHB families, an increase in SES means an increase in experience [95,96,97,98,99] and vulnerability [100] to discrimination. This might be because high SES NHB families are more likely to be surrounded by NHW families, which means a higher level of exposure to discrimination [95,96]. Needless to say, a high level of discrimination means undesired outcomes and reduced gains of SES [98,100,101].

Residential segregation results in differences in NHB and NHW environmental and contextual exposures. Due to segregation, school options are different for high SES NHB and HW families. As a result, children of high SES NHB families attend highly segregated schools with low resources [62,63,102]. That means there are differential effects of SES on education and schooling of NHW and NHB. While high SES NHW youth attend schools in suburban areas with more funding and higher-quality teachers, NHBs should go to schools that are of lower quality [103].

While lower SES of NHBs is one type of disadvantage, MDRs reflect another class of disadvantage [52,53]. While the first one is about a lack of access to SES resources, MDRs are reflective of unequal outcomes despite similar access to SES. Thus, researchers and policymakers should not only address inequality in SES, but they should also address inequality in the returns of SES. Unfortunately, NHB families are at a double disadvantage because they are affected by low SES and low return of the existing SES resources [52,104]. 

Multilevel economic, psychological, and societal mechanisms may be involved in explaining racial and ethnic gaps in the returns of parental education [52,104]. MDRs may be due to racism across multiple societal institutions and social structures [52,104]. Racial/ethnic prejudice interferes with the processes that are needed to gain benefits from available SES resources [105,106,107]. MDRs of educational attainment may be in part due to a history of childhood poverty [108]. The current study, however, did not explore societal and contextual processes that could explain such MDRs.

NHB families are more likely to stay in poor neighborhoods despite high SES. Highly educated NHBs are more likely to stay poor than NHWs [66,109]. Similarly, NHB families from high SES backgrounds may remain at risk of environmental exposures than NHWs with similar SES [95,96,98,110,111,112,113]. Similarly, high SES NHB youth spend time with peers with higher risk and behavioral problems than NHWs with the same SES [54,91].

The implications of these MDRs are that it is not merely the access to SES, but also the degree to which SES can result in outcomes that should be addressed. Interventions should target the societal, social, environmental, and structure processes that generate MDRs. We argue that the solutions to MDRs-related disparities are different from those due to low SES. While the solution to disparities due to the gap in SES is to increase NHBs’ access to SES resources, the remedy to MDRs-related inequalities to empower NHBs so they can more efficiently translate their SES to outcomes. The latter solution requires policies and programs that go beyond access and address structural and environmental factors. For the latter, there is a need to equalize the life conditions of NHBs and NHWs.

## 6. Implications

The results may still be preliminary and need further evaluation. If these results are replicated by other datasets and samples, then there is a need for an intervention. It can be argued that NHB parents may require some additional help to mobilize their educational resources. NHB parents may benefit from programs that help with the employability of highly educated NHB parents. Schools in urban areas may benefit from additional resources and an enhanced quality of teachers. After school programs are some example solutions that may compensate reduced IC of NHB youth across SES levels. Interventions may also specifically target the developmental needs of NHB youth across all SES levels. Clinicians should be aware that some of the needs of NHB youth remain high across all SES levels. So, clinicians should be informed that some of the benefits and advantages that NHW youth receive from high SES may not be relevant in NHB families. Low attention may also be diagnosed and treated in NHB families across SES levels. Policymakers may also go beyond family SES and address structural factors that hinder NHB families from mobilizing their SES resources, particularly educational attainment, for securing outcomes.

## 7. Limitations and Strengths

Each study comes with specific types of methodological limitations. As our data were cross-sectional, we cannot draw any causal inferences between race, parental educational attainment, and youth IC. Similarly, this study only tested the MDRs of parental educational attainment. Other research may test if MDRs also hold for other SES indicators such as parental occupational prestige, income, wealth, and neighborhood SES. These investigations are important because associations between various SES indicators depend on race/ethnicity. Finally, this study only described the MDRs and did not explore contextual factors that could potentially explain the observed MDRs. 

To list the study strengths, we can refer to the national scope, multi-center nature, large sample size, large sample of NHBs, and focusing on multiplicative rather than additive effects of SES and race. We can also refer to the application of multiple IC measures (the stop-signal task and CBCL) as another strength of the study. It is important to validate the youth IC task performance with the CBCL parent ratings for several reasons. First, in a clinical setting, most clinicians can easily utilize data of CBCL and measure IC using parents’ ratings. However, in many clinical and school settings, collecting data on IC using time-consuming tasks may be difficult. This is also applicable to scaling up the study results. While public health programs and interventions may apply self-reported measures of attention as a screening tool, it is unlikely that large programs can access laboratory measures of attention. So, our multiple measures of IC make our results applicable to a large-scale intervention. Finally, any study of racial differences should always cross-validate their measure. We found the IC similarly correlate with clinical presentation (parental report) of IC, so our IC construct was reflecting the same concept and is similarly applicable to NHW and NHB youth. In other terms, our lab-based measure of IC refers to the very same understanding of parents about their youth behavioral and attention problems. So, the results are similarly relevant to NHB and NHW families. This approach helps us rule out that the observed racial differences are simply an artifact and due to racial variation in measurement properties.

## 8. Conclusions

When compared to NHWs, NHB youth show lower parental educational attainment and parent reported IC. This adversity is compound by another more profound and systemic adversity, which reflects a weaker effect of parental educational attainment and youth IC. As a result of the latter disadvantage, NHB youth show low IC even when they have highly educated parents. That means some of the inequalities in IC remains across all SES levels. In other terms, racial and ethnic inequalities in IC show a spill-over effect in middle-class people. As a result of high IC, NHB youth with highly educated parents may remain at risk of social, emotional, and behavioral problems. 

## Figures and Tables

**Table 1:** Data overall and by race/ethnicity (*n* = 4188).

	All		NHWs		NHBs	
	***n***	**%**	***n***	**%**	***n***	**%**
**Ethnicity**						
NHWs	2985	71.3	2985	100.0	-	-
NHBs	1203	28.7	-	-	1203	100.0
**Sex**						
Male	2026	48.4	1426	47.8	600	49.9
Female	2162	51.6	1559	52.2	603	50.1
Marital Status *						
Other	1103	26.5	399	13.4	704	60.0
Married	3054	73.5	2584	86.6	470	40.0
	**Mean**	**SD**	**Mean**	**SD**	**Mean**	**SD**
Age (Year)	9.45	0.50	9.45	0.50	9.46	0.51
Parental Educational Attainment *	16.90	2.42	17.58	1.99	15.20	2.55
CBCL Anxiety Depressed *	2.62	3.15	2.74	3.19	2.31	3.05
CBCL Withdraw Depressed *	1.00	1.71	0.93	1.63	1.16	1.89
CBCL Somatic Complaints	1.48	1.91	1.51	1.89	1.42	1.98
CBCL Social Problems *	1.58	2.29	1.46	2.22	1.88	2.44
CBCL Thought Problems *	1.66	2.25	1.71	2.22	1.54	2.32
CBCL Rule Breaking *	1.25	1.94	1.07	1.69	1.70	2.40
CBCL Attention Problems *	5.39	5.40	5.24	5.24	5.75	5.77
CBCL Aggressive Behaviors *	3.44	4.58	3.27	4.32	3.85	5.15
14 SST-based IC	0.54	0.11	0.54	0.10	0.54	0.12

Child Behavior Checklist (CBCL); Inhibitory Control (IC); Non-Hispanic Black (NHB); non-Hispanic White (NHW); Stop-Signal Task (SST); Standard Deviation (SD); * *p* < 0.05.

**Table 2:** Correlations between study variables (*n* = 4188).

	1	2	3	4	5	6	7	8	9	10	11	12	13	14
All														
1 Race (NHBs)	1	−0.02	0.01	−0.48 **	−0.45 **	−0.06 **	0.06 **	−0.02	0.08 **	−0.03 *	0.15 **	0.04 **	0.06 **	0.02
2 Sex (male)		1	0.03	0.01	−0.00	0.01	0.05 **	−0.04 *	0.05 **	0.09 **	0.11 **	0.15 **	0.10 **	−0.11 **
3 Age (Years)			1	−0.01	−0.02	0.01	.042 **	0.02	0.00	0.01	0.02	0.00	0.00	−0.03
4 Family Structure (married)				1	0.37 **	−0.02	−0.11 **	−0.05 **	−0.11 **	−0.03 *	−0.17 **	−0.10 **	−0.11 **	−0.02
5 Parental Educational Attainment (Years)					1	0.00	−0.11 **	−0.04 *	−0.13 **	−0.07 **	−0.18 **	−0.10 **	−0.11 **	0.01
6 Child Behavior Checklist (CBCL) Anxiety Depressed						1	0.57 **	0.48 **	0.61 **	0.60 **	0.40 **	0.56 **	0.56 **	−0.02
7 CBCL Withdraw Depressed							1	0.40 **	0.55 **	0.49 **	0.38 **	0.47 **	0.49 **	−0.01
8 CBCL Somatic Complaints								1	0.43 **	0.45 **	0.29 **	0.44 **	0.39 **	−0.03 *
9 CBCL Social Problems									1	0.61 **	0.56 **	0.70 **	0.68 **	−0.02
10 CBCL Thought Problems										1	0.52 **	0.73 **	0.64 **	−0.06 **
11 CBCL Rule Breaking											1	0.67 **	0.75 **	−0.04 **
12 CBCL Attention Problems												1	0.76 **	−0.05 **
13 CBCL Aggressive Behaviors													1	−0.04 *
14 IC														1
**NHWs**														
2 Sex (male)		1	0.03	−0.02	−0.03	0.01	0.06 **	−0.05 **	0.04 *	0.08 **	0.10 **	0.15 **	0.10 **	−0.13 **
3 Age (Years)			1	0.01	−0.03	0.03	0.05 **	0.03	−0.00	0.02	0.00	0.00	0.01	−0.06 **
4 Family Structure (married)				1	0.17 **	−0.07 **	−0.12 **	−0.09 **	−0.11 **	−0.07 **	−0.13 **	−0.11 **	−0.10 **	0.04 *
5 Parental Educational Attainment (Years)					1	−0.08 **	−0.12 **	−0.12 **	−0.16 **	−0.13 **	−0.18 **	−0.15 **	−0.14 **	0.06 **
6 CBCL Anxiety Depressed						1	0.57 **	0.47 **	0.59 **	0.57 **	0.39 **	0.53 **	0.55 **	−0.00
7 CBCL Withdraw Depressed							1	0.38 **	0.53 **	0.48 **	0.40 **	0.45 **	0.48 **	−0.03
8 CBCL Somatic Complaints								1	0.42 **	0.43 **	0.30 **	0.44 **	0.38 **	−0.03
9 CBCL Social Problems									1	0.60 **	0.56 **	0.68 **	0.67 **	−0.03
10 CBCL Thought Problems										1	0.51 **	0.72 **	0.61 **	−0.05 **
11 CBCL Rule Breaking											1	0.64 **	0.74 **	−0.04 *
12 CBCL Attention Problems												1	0.73 **	−0.04 *
13 CBCL Aggressive Behaviors													1	−0.03
14 IC														1
**NHBs**														
2 Sex (male)		1	0.03	0.03	0.02	0.03	0.02	−0.01	0.09 **	0.12 **	0.14 **	0.17 **	0.12 **	−0.06 *
3 Age (Years)			1	−0.01	0.02	−0.01	0.03	0.01	0.01	0.01	0.04	−0.00	−0.00	0.04
4 Family Structure (married)				1	0.26 **	−0.01	−0.06 *	−0.02	−0.05	−0.03	−0.09 **	−0.05	−0.08 **	−0.08 **
5 Parental Educational Attainment (Years)					1	0.07 *	−0.05	0.07 *	−0.01	−0.01	−0.07 *	0.01	−0.03	−0.04
6 CBCL Anxiety Depressed						1	0.59 **	0.51 **	0.70 **	0.67 **	0.48 **	0.64 **	0.62 **	−0.04
7 CBCL Withdraw Depressed							1	0.44 **	0.58 **	0.52 **	0.34 **	0.51 **	0.50 **	0.01
8 CBCL Somatic Complaints								1	0.46 **	0.49 **	0.31 **	0.45 **	0.42 **	−0.04
9 CBCL Social Problems									1	0.65 **	0.58 **	0.72 **	0.70 **	−0.01
10 CBCL Thought Problems										1	0.57 **	0.75 **	0.69 **	−0.07 *
11 CBCL Rule Breaking											1	0.72 **	0.79 **	−0.05
12 CBCL Attention Problems												1	0.80 **	−0.07 *
13 CBCL Aggressive Behaviors													1	−0.04
14 IC														1

* *p* < 0.05; ** *p* < 0.01.

**Table 3:** Summary of linear regressions by race/ethnicity (*n* = 4183).

	Model 1 NHWs			Model 2 NHBs		
	Beta	SE	*p*	Beta	SE	*p*
Sex (Male)	−0.13	−0.03	<0.001	−0.06	−0.01	0.047
Age	−0.06	−0.01	0.002	0.04	0.01	0.165
Married household	0.03	0.01	0.139	−0.08	−0.02	0.012
Parental Educational Attainment	0.05	0.00	0.003	−0.02	0.00	0.503
Constant		0.60	<0.001		0.48	<0.001

Unstandardized Regression Coefficient (B); Confidence Interval (CI); Standard Error (SE); Non-Hispanic Black (NHB); Outcome: Stop-Signal Task (SST)-based Inhibitory Control (IC).

**Table 4:** Summary of linear regressions overall (*n* = 4183).

	Model 1 Main Effects			Model 2 Interaction Effects		
	Beta	SE	*p*	Beta	SE	*p*
Race/Ethnicity (NHBs)	0.02	0.00	0.315	0.35	0.08	0.001
Sex (Male)	−0.11	−0.02	<0.001	−0.10	−0.02	<0.001
Age	−0.02	−0.01	0.118	−0.02	0.00	0.131
Married household	−0.02	0.00	0.300	−0.02	0.00	0.386
Parental Educational Attainment	0.03	0.00	0.097	0.07	0.00	0.001
Parental Educational Attainment × NHBs				−0.31	0.00	0.002
Constant		0.58	<0.001		0.54	<0.001

Unstandardized Regression Coefficient (B); Confidence Interval (CI); Standard Error (SE); Non-Hispanic Black (NHB); Outcome: Stop-Signal Task (SST)-based Inhibitory Control (IC).
